# SERS of Bottled Water: Automated PET Nanoplastic Detection and Quantification

**DOI:** 10.1021/acs.analchem.6c04234

**Published:** 2026-09-26

**Authors:** Ion Nesterovschi, Ioana Cârdan, Karlo Maškarić, Anamaria Couţi, Lucian Barbu-Tudoran, Simona Cinta-Pinzaru

**Affiliations:** † Biomolecular Physics Department, 280365 Babeş-Bolyai University, Kogălniceanu 1, Cluj-Napoca RO-400084, Romania; ‡ Institute for Research, Development and Innovation in Applied Natural Sciences, Babes-Bolyai University, Fântânele 30, Cluj-Napoca RO-400327, Romania; § National Institute for Research and Development of Isotopic and Molecular Technologies, Cluj-Napoca 400293, Romania

## Abstract

Nanoplastics represent an emerging contaminant of growing environmental and public health concern, with polyethylene terephthalate (PET) being on topic due to its dominant use in beverage packaging. This study presents a SERS approach for the detection and quantification of PET nanoplastics in commercial bottled water, combining SERS with colloidal silver nanoparticles (AgNPs) and commercial solid SERS substrates, with automated Pearson correlation spectral classification. Mapping SERS samples of PET-bottled water combined with a Python identification algorithm enabled the detection of PET nanoplastics, while SEM-EDX analysis confirmed the presence of nanoscale carbon–oxygen particles consistent with PET stoichiometry. Beyond PET, phthalocyanine pigment nanoparticles, likely originating from bottle package, and inorganic residues including calcite, gypsum, and sodium nitrate were simultaneously identified, demonstrating the multianalyte capability of the approach. Using colloidal AgNPs, the estimated PET nanoplastic concentration was 17.25 ± 1.6 million particles per 0.5 L bottle (34.5 ± 3.2 million particles/L), comparative with values reported by nanoparticle tracking analysis (NTA) and stimulated Raman scattering (SRS) microscopy in comparable studies. The method requires minimal sample preparation, limited to concentration by evaporation, without digestion or extraction steps, and preliminary results can be obtained within a few hours. These findings establish SERS combined with automated spectral analysis as a practical and sensitive screening tool for nanoplastic monitoring in drinking water, reporting particle concentrations directly in the number of particles per liter and with potential applicability to broader water quality analysis.

## Graphical abstract

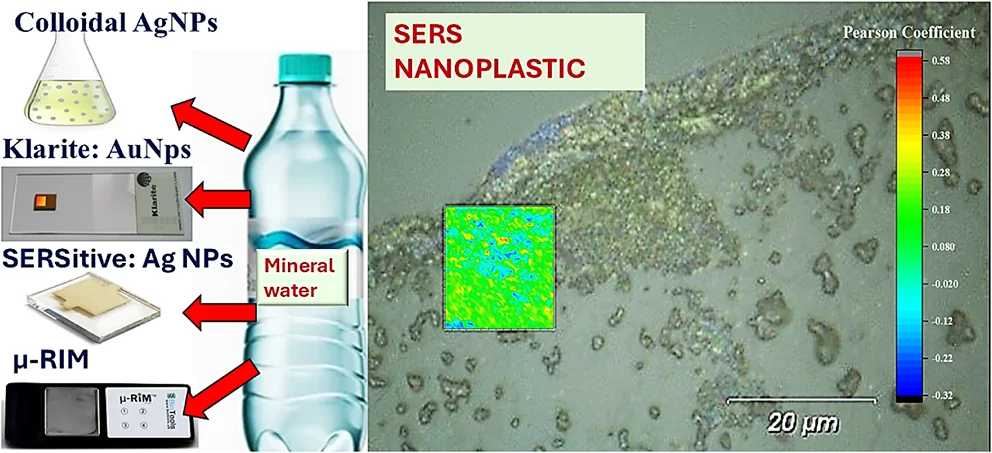

## Introduction

Plastic pollution is one of the most crucial environmental issues of our time, with global plastic production surpassing 400 million tons annually, and a significant portion of this material ends up in aquatic ecosystems.^1^ Over time, macro- and microplastics undergo degradation due to environmental factors such as UV radiation, mechanical factors, and microbial interaction, eventually breaking down into nanoscale particles known as nanoplastics (NPs), which are smaller than 1000 nm.^2–4^ These small fragments represent a serious concern not only for ecosystems but also for human health, as their small size allows them to penetrate biological barriers, interact with cell elements, and potentially induce oxidative stress, inflammation, and endocrine disruption.^5,6^

Recent studies have detected micro- and nanoplastics in various parts of the human body, including blood,^7^ placenta,^8^ and lungs, with the most recent and concerning findings indicating their presence in the brain.^9^ Although their presence has been demonstrated, current detection and quantification methods—particularly for nanoplastics, in complex matrices, require improvements toward faster, simpler, and less invasive approaches.

From this perspective, the detection of polyethylene terephthalate (PET) nanoplastics is particularly relevant because of the wide use of PET in food packaging and especially in bottled water. These containers are exposed to various environmental conditions such as light, temperature fluctuations, mechanical stress, and prolonged contact with water from the inside of the bottle, all of which can promote polymer fragmentation and particle release.^10^ Thus, several studies have already reported the presence of microplastics in bottled drinking water, with PET identified as a dominant polymer originating from the packaging material itself, confirming bottled water as a direct source of plastic contamination.^11,12^ Consequently, PET nanoplastics may represent one of the major contributors to human nanoplastic exposure from bottled water, raising critical concerns for public health.^13^

In this regard, the detection and quantification of PET nanoplastics in drinking water are critical for understanding the exposure risks.^14,15^

Traditionally, analytical techniques such as gas chromatography-mass spectrometry (GC-MS) and pyrolysis-GC-MS have been employed to identify plastic polymers in nanoplastics.^16,17^ These methods, while effective in identifying polymer type, are destructive by nature and do not provide information about particle size. To solve the limitations of destructive techniques, nondestructive optical methods such as Fourier transform infrared (FTIR) spectroscopy are used to identify plastic particles based on their vibrational spectra.^18^ However, the spatial resolution of FTIR is limited to particles larger than 20 μm, being inadequate for nanoplastics. Techniques such as atomic force microscopy-infrared (AFM-IR) spectroscopy offer a better resolution, down to 10 nm, but are time-consuming, and the sample preparation is too complex.^19^ In contrast, Raman spectroscopy can be a powerful technique for identifying plastics due to its ability to provide molecular fingerprints with high spatial resolution, down to 1 μm, and minimal sample preparation.^14,20^ However, Raman spectroscopy suffers from weak signal intensity, which is the main limitation when analyzing nanoplastics.

To fix the sensitivity limitation for classical Raman technique, more advanced methods are used for NPs’ detection such as hyperspectral stimulated Raman scattering (SRS) imaging and surface-enhanced Raman scattering (SERS).^21–23^ SERS is one of the most promising techniques for the detection of environmental nanoplastics, as it relies on localized surface plasmon resonances generated by nanostructured metallic surfaces, which amplify the Raman scattering signal of nearby molecules by several orders of magnitude.^24,25^ These nanostructured metallic surfaces can be categorized into colloidal and solid SERS substrates, each with distinct fabrication methods, advantages, and limitations.^26^ Liquid SERS substrates, typically composed of colloidal silver or gold nanoparticles, are prepared via chemical reduction methods such as citrate or borohydride reduction.^27^ These colloids offer high enhancement factors due to the formation of “hot spots” between aggregated nanoparticles. However, they suffer from batch-to-batch variability and limited stability in complex samples.^28,29^ In contrast, solid SERS substrates are fabricated by using various techniques such as advanced lithography,^30,31^ colloidal lithography,^32,33^ physical vapor deposition, and electrochemical or chemical deposition, among others.^34^ These substrates offer enhanced reproducibility, spatial uniformity, and chemical stability, making them well-suited for routine and field applications. However, their enhancement factors are generally lower than those of colloidal systems, and fabrication costs can be higher.^35^

Over the past few years, SERS has been successfully employed to detect a range of polymer types, including polystyrene, polyethylene, and polyethylene terephthalate.^36–38^ Recent studies have provided detailed insights into the plasmonic amplification mechanisms occurring on nanoplastic particles when solid SERS substrates are used, highlighting the critical influence of substrate morphology as well as intrinsic characteristics of the nanoplastics, such as particle size and shape.^39^ Engineered gold substrates with ring-shaped nanogaps have also demonstrated the detection of 50 nm PS nanoparticles at 1 μg/mL, along with the successful identification of polypropylene (PP) in drinking water and polyethylene terephthalate (PET) in wash water from infant feeding bottles.^36^ These improvements demonstrate the ability of SERS to detect nanoplastics at environmentally relevant sizes and concentrations, motivating further investigation into the presence of polypropylene (PP), polyethylene (PE), and PET nanoparticles in commercially available bottled water.

Several recent studies on commercial bottled water^15,38,40^ have reported the presence of polypropylene (PP), polyethylene (PE), and polyethylene terephthalate (PET) nanoplastics, with PET reaching the highest concentrations (4700 ng/L), which represents a significant exposure to people, together with leached plastic additives.^40^ However, most of these investigations rely primarily on mass-spectrometry techniques, which provide accurate polymer identification but, as discussed earlier, require extensive sample preparation. Complementary work by Zhang et al.^38^ used the SERS technique based on gold triangular array solid substrates to identify nanoplastics in bottled water. While this study demonstrated the potential of SERS for polymer detection, quantitative results were derived from nanoparticle tracking analysis (NTA) rather than directly from SERS measurements, reporting a concentration of approximately 10^8^ particles/mL. In contrast, the present study makes progress in this topic by using SERS mapping with correlation-based spectral analysis to identify and estimate the number of nanoplastics from Raman data.

Unlike previous SERS studies that introduced polymer nanoparticles of known concentration into clean matrices or derived particle counts from nanoparticle tracking analysis rather than directly from SERS data,^38^ the present study detects and counts naturally occurring nanoplastics from a commercially available bottled water product using SERS mapping as the primary identification and counting tool without any intentional sample spiking. The present study explores two SERS-based substrates for the detection and quantification of PET nanoplastics in bottled water. The first type of substrate uses colloidal silver nanoparticles (AgNPs) as the active-SERS substrate, while the second is based on commercially available solid SERS substrates. The two SERS methodologies used a Pearson correlation algorithm and a plastic Raman database^41^ aiming for an efficient identification of PET nanoplastics alongside other organic or inorganic molecules. Complementarily, SEM-EDX analysis confirmed the presence of nanoscale carbon–oxygen particles consistent with PET composition. Alongside PET, we also identified additional nanoparticles such as phthalocyanine pigments, possibly originating from package like bottle cap or bottle itself. The analysis also revealed inorganic residues such as calcite, gypsum, and sodium nitrate. The PET nanoparticle concentration was estimated at approximately 17.25 ± 1.6 million per 0.5 L water bottle, confirming that SERS with automated spectral analysis enables nondestructive detection and quantification of nanoplastics in bottled water.

## Materials and Methods

### Water Sample Preparation

Bottled water samples (0.5 L each) were randomly purchased from a local retailer. According to the manufacturer, the product is “Natural Still Mineral Water DORNA—White Spring (Izvorul Alb),” originating from Suceava County, Romania. Its chemical composition, based on regulatory analysis Series N, No. 016774 (31.08.2012; SR 4450/97), is reported as follows (mg/L): Ca^2+^ 63.66, Mg^2+^ 1.2, Na^+^ 0.92, K^+^ 0.4, Cl^–^ 4.8, NO_3_^–^ 4.13, with a pH of 7.49 and a dry residue of 175 mg/L at 180 °C. The product is certified microbiologically pure and supplied in 0.5 L plastic packaging and widely available in the market.

For analysis, the entire 0.5 L sample was transferred into a cleaned Berzelius beaker and evaporated at 70 °C for 66 h until a final volume of 10 mL was obtained. This temperature is below the melting and thermal degradation thresholds of PET and other common packaging polymers and is therefore not expected to induce polymer fragmentation or nanoplastic formation. The resulting concentrated sample was subsequently used for SERS measurements.

### SERS Measurements Using Silver Colloidal Nanoparticles

Colloidal silver nanoparticles (AgNPs) were synthesized following the classical Lee–Meisel method.^27^ After synthesis, the AgNPs were characterized by UV–vis spectroscopy for their absorbance and full width at half-maximum (fwhm). Additionally, AgNPs were evaluated for their bulk Raman and SERS responses using well-known molecules (crystal violet and methylene blue), where the nanoparticles showed reproducible spectral signals, consistent with previous reports.^38^ The SERS sample preparation was carried out by mixing 1 mL of the AgNP colloid with 20 μL of the concentrated water sample obtained after evaporation. A 2 μL sample of this mix was then drop-cast onto an aluminum foil-coated glass microscope slide and allowed to dry at room temperature. The aluminum layer served as a reflective support, enhancing the backscattered Raman signal and thereby improving SERS intensity. Raman measurements were performed at the edge of the dried droplet, known as the “coffee-ring”. The measurements are based on Raman mapping of this region with a grid size of 0.3 μm, which approaches the diffraction-limited spatial resolution of the system (0.33 μm for 532 nm excitation with a 0.98 NA objective).

Raman spectra were acquired using a Renishaw InVia Raman microscope system equipped with a Leica optical microscope and a thermoelectrically cooled CCD Centrus detector (Renishaw), providing high quantum efficiency and low detector noise, which represent an advantage for large-area mapping experiments requiring thousands of spectra at low laser power and short integration times. Spectral mapping was conducted using a 532 nm laser excitation source with a power output of 2 mW and an integration time of 0.1 s per point, using an objective of 100× magnification (NA 0.98). The spectral acquisition range was set between 100 and 1800 cm^–1^ to capture characteristic vibrational modes of PET and other contaminants.

### Blank Measurements

Blank measurements were performed following the same protocol as for the evaporated bottled water sample, where the bottled water was replaced by 20 μL of ultrapure distilled water mixed with 1000 μL of the colloidal AgNPs. A total of approximately 15000 Raman spectra were acquired across an area of 1350 μm^2^, distributed over seven independent Raman mapping acquisitions from different dried AgNP-distilled water droplets. The Pearson correlation threshold was applied to all blank spectra to identify false positives.

### SERS Measurements Using Commercial Solid Substrates

Three commercial SERS substrates, a) standard Klarite (a type of commercially available, single-use slide sold by D3 Technologies Ltd., Glasgow, U.K.), b) SERSitive (SERSitive, Kasprzaka 44/52, 01-224, Warsaw, Poland), and c) μ-RIM (a highly reflective stainless steel substrate from BioTools, Woburn, MA 01801, USA), were purchased and used in the present study for the spectroscopic analysis of the concentrated water samples. On each substrate, a volume of 1 μL of concentrated water was drop-coated and after drying, the Raman analysis was performed using the laser excitation at 532 nm from a cobalt DPSS laser. All SERS spectra were acquired using the same Renishaw InVia Raman system with an objective magnification of 100× (NA 0.98). Spectral mapping was performed by scanning several random areas of 10 × 10 μm on each solid substrate with a scanning step of 0.7 μm. The acquisition parameters used for mapping were 0.5 s integration time, 2 mW power, and 100× objective magnification.

### Data Processing

Spectral data were processed using Python 3.11 and OriginPro 2021b (OriginLab Corporation, Northampton, USA). Denoising was performed using the Anscombe transformation, followed by baseline correction with the Improved Asymmetric Least Squares algorithm. The nanoplastic identification algorithm involves comparing each processed Raman spectrum within the mapping data to the reference spectra of common polymers, such as polyethylene terephthalate (PET), polyethylene (PE), polyamide (PA), polyvinyl chloride (PVC), polypropylene (PP), and polystyrene (PS). This comparison is conducted using the Pearson correlation coefficient to evaluate spectral similarity. The Pearson correlation coefficient r between a measured spectrum and a reference spectrum is defined as

$$r=\frac{\overset{n}{\underset{i=1}{\sum }}\left(x_{i}-\mathrm{x̅}\right)\left(y_{i}-\mathrm{y̅}\right)}{\sqrt{\overset{n}{\underset{i=1}{\sum }}{\left(x_{i}-\mathrm{x̅}\right)}^{2}\cdot \overset{n}{\underset{i=1}{\sum }}{\left(y_{i}-\mathrm{y̅}\right)}^{2}}}$$

where x_i_ and y_i_ are the intensities of the measured and reference spectra at wavenumber i, x̅ and y̅ are their respective means, and n is the number of spectral data points. The coefficient ranges from −1 to +1, where +1 indicates perfect linear correlation between the two spectra, 0 indicates no correlation, and values approaching −1 indicate an inverse spectral relationship. For polymer identification, only values close to +1 are meaningful, as they reflect spectral similarity between the measured signal and the reference spectrum. The correlation was calculated over the spectral range 800–1800 cm^–1^, which includes all diagnostic vibrational bands of PET and the other polymers in the database. Prior to correlation, all spectra were intensity-normalized to their maximum value within this range to ensure that the coefficient reflects spectral shape similarity independently of absolute signal intensity. Although the Pearson correlation coefficient is sensitive to changes in relative band intensities, peak positions, and fwhm, the Pearson threshold directly represents this sensitivity; thus, the spectra falling below the threshold either correspond to a chemically different molecule or represent a polymer that has undergone sufficient chemical modification through degradation or oxidation such that its molecular identity can no longer be precisely assigned as PET or any other compared polymer.

The threshold value to confirm nanoplastic identification was established using three complementary approaches. First, the PET reference Raman spectrum was mathematically mixed with Raman spectra of other polymers and pigments obtained from an open spectral database.^42^ The mixing procedure consisted of the point-by-point addition of the intensities at the same wavenumbers. Each mixed spectrum was then correlated with the pristine PET spectrum by using the Pearson coefficient. The lowest Pearson’s value obtained from these comparisons (0.7) was selected as a potential identification threshold (Table S1 and S2).

Second, an experimental false-positive assessment was performed by using the blank AgNP samples. Approximately 15000 Raman spectra acquired from 7 independent Raman mappings were correlated with the PET reference spectrum using the potential threshold identified from the spectral mixing analysis. No false-positive PET identifications were observed across any of the mapping acquisitions, confirming that neither citrate nor any other AgNP spectral contribution produced a Pearson correlation coefficient exceeding this threshold under the measurement conditions.

Third, artificial spectral modifications were introduced to simulate the PET degradation effects. PET nanoplastics are subject to change of the ester bonds, surface oxidation, and UV-induced crystallinity changes in the environment. These degradation pathways directly affect the PET Raman spectrum; for example, the intensity ratio I_1729_/I_1615_ has been shown to depend on crystallinity degree.^43,44^

Thus, the relative band intensities were varied by 50% and band positions were shifted by 5 cm^–1^ to mimic potential structural changes reported also macro-/microplastics degradation studies.^14,23,45–47^ Each modified spectrum was correlated with the pristine PET spectrum, and the lowest Pearson coefficient obtained was again 0.7 (Figure S1).

The merging of all three approaches toward the same Pearson value confirms that 0.7 represents a reliable threshold for the identification of PET nanoplastics.

### Scanning Electron Microscopy Measurements

Scanning electron microscopy (SEM) measurements on dried, concentrated water droplets were done using a Hitachi SU8230 (Hitachi High-Tech Co., Ltd., Japan) system. SEM measurements were complemented by energy-dispersive X-ray (EDX) spectroscopy analyses.

### Dynamic Light Scattering Measurements

Particle size characterization of the silver nanoparticles and particles present in the evaporated bottled water samples was performed using dynamic light scattering (DLS). Measurements were carried out with a Vasco Kin instrument (Cordouan Technologies) equipped with a 635 nm laser operating at 3 mW. Each measurement was recorded for an acquisition time of 30 s.

### Preparation of PET Micro- and Nanoplastic Reference Suspension

A transparent, colorless PET bottle was cut into approximately 1 cm × 1 cm fragments and washed with ethanol and ultrapure water to remove surface contaminants before milling. Mechanical milling was performed using a Retsch Mixer Mill MM 400, equipped with zirconium oxide grinding balls operating at 30 Hz for 30 min. The resulting powder was collected and suspended in ethanol. To isolate the submicron fraction, the suspension was passed through a 2 μm cellulose membrane filter. The filtrate was retained for further characterization and used as the PET reference material. The hydrodynamic size distribution of the filtered suspension was determined by dynamic light scattering (DLS) to confirm the presence of particles in the nanoplastic size range. The SERS Raman spectra of the filtrate were acquired to confirm spectral identity with the PET reference spectrum used in the Pearson correlation analysis.

## Results

### Colloidal AgNP Characterization

The colloidal AgNP substrate was characterized by UV–vis absorption spectroscopy and dynamic light scattering (DLS) before its use as the SERS substrate.

Time-resolved DLS (Figure 1A) showed a progressive shift from the initial AgNP size of 68 nm (dashed line) toward a population at approximately 200 nm after 1 h, confirming nanoparticle aggregation driven by the ionic content of the evaporated water. At 45 min, the main DLS band splits into two, indicating the presence of the primary AgNPs and a newly formed aggregate. Afterward, the dominant population reaches its maximum size of approximately 200 nm at 1 h 15 min, after which it shifts back toward smaller sizes at later time points, consistent with the sedimentation of the largest aggregates from the measured volume.

**Figure 1:**
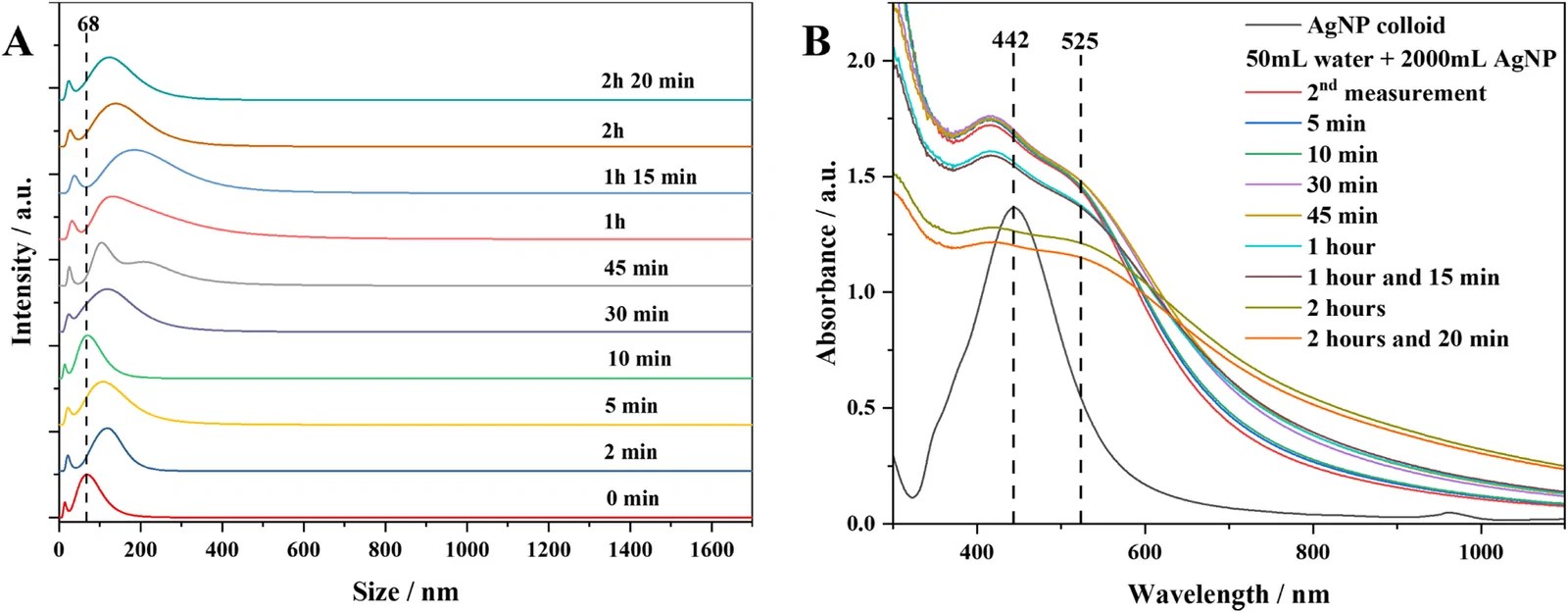
Characterization of colloidal AgNP and its interaction with evaporated bottled water. (A) Stacked DLS size populations recorded at successive time intervals (0 min to 2 h 20 min, bottom to top), with the dashed line marking the initial AgNP size; (B) UV–vis absorption spectra recorded at time intervals after mixing AgNPs with evaporated bottled water, showing progressive broadening and a redshift of the plasmon band from 442 nm (dashed line), with the emergence of a shoulder at 525 nm (dashed line).

This aggregation was also independently confirmed by UV–vis spectroscopy (Figure 1B), which revealed the characteristic plasmon absorption of the blank AgNP colloid at 442 nm and showed progressive broadening of the plasmon band with the appearance of a shoulder at approximately 525 nm, becoming more pronounced at later time points, consistent with the ionic content of the evaporated water driving aggregate formation.

### Identification of PET Nanoplastics in the Reference Suspension Using AgNP Colloidal Substrates

DLS characterization of the filtered milled PET suspension confirmed the presence of particles in the nanoplastic size range, with a dominant population centered at 291 nm (Figure 2A). To validate the identification protocol, a 2 μL droplet of the milled PET suspension mixed with colloidal AgNPs (20 μL of PET NP with 1 mL of AgNP colloid) was drop-cast onto aluminum foil and analyzed by Raman mapping at 532 nm excitation. A representative SERS spectrum extracted from a PET-positive mapping point is shown in Figure 2B, together with the blank AgNP spectrum and the PET reference Raman spectrum.

**Figure 2:**
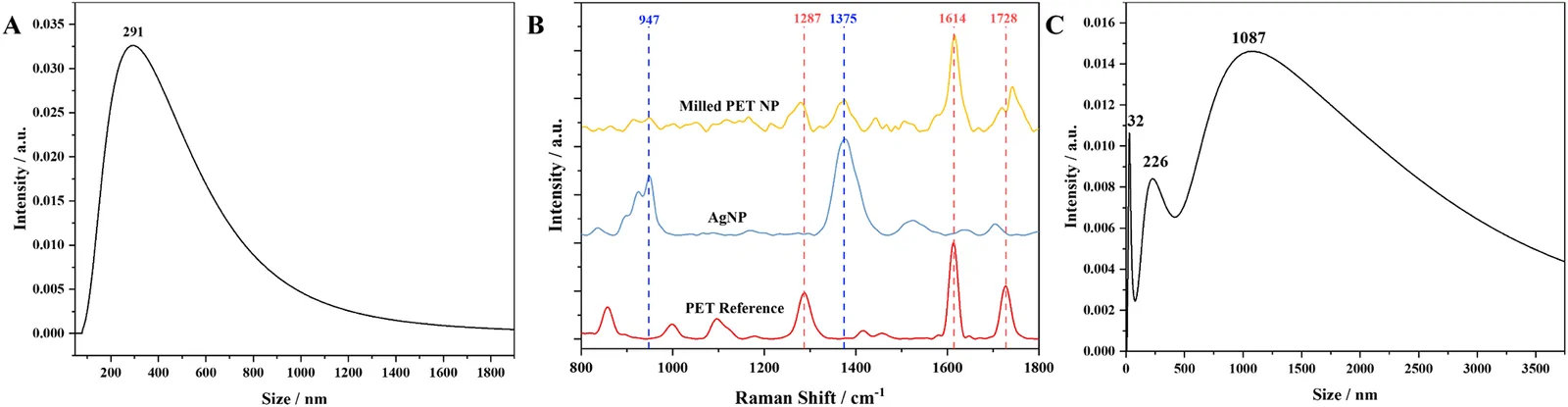
(A) Hydrodynamic size distribution of the milled PET reference suspension by DLS, confirming a dominant nanoplastic population at 291 nm. (B) Representative SERS spectrum from a PET-positive mapping point in the dried AgNP-milled PET suspension (yellow), compared to the blank AgNP spectrum (blue) and PET reference Raman spectrum (red). Specific PET bands at 1287, 1614, and 1728 cm^–1^ (red dashed lines) and citrate-related AgNP bands at 947 and 1375 cm^–1^ (blue dashed lines) are indicated. Laser excitation: 532 nm. (C) Hydrodynamic size distribution of the evaporated bottled water sample by DLS, showing three populations at 32, 226, and 1087 nm, attributed to sub-50 nm salt aggregates, nanoplastics, and large nanoplastic aggregates, respectively.

The blank AgNP spectrum (blue line) shows two intense bands at 947 and 1375 cm^–1^, assigned to citrate adsorbed on the nanoparticle surface in its role as a reducing and capping agent in the Lee–Meisel synthesis;^48,49^ these citrate bands partially overlap with the diagnostic PET bands, which is addressed through the Pearson correlation threshold as described in the Data Processing section.

The identified spectrum shows the three diagnostic PET bands at 1287, 1614, and 1728 cm^–1^, confirming PET identification, alongside bands at 947 and 1375 cm^–1^ from the citrate adsorbed on the AgNP surface. PET identification was performed using the same Pearson correlation algorithm and threshold applied to the bottled water sample, confirming a positive identification in the milled PET reference suspension. Across four independent mapping acquisitions with a total area of 990 μm^2^, three PET-positive points were identified. Quantification was not performed as the purpose of this experiment was to validate the detection algorithm.

These results validate the identification protocol against real PET nanoparticles, confirming that the Pearson correlation algorithm correctly classifies PET in the SERS measurements, despite additional Raman bands from the AgNP substrate.

### SERS of Bottled Water Using AgNP Colloidal Substrates

DLS characterization of the evaporated bottled water revealed three size populations at 32, 226, and 1087 nm (Figure 2C), likely corresponding to salts after evaporation, nanoplastics, and microplastic particles or nanoplastic aggregates. The 1087 nm population is attributed to large nanoplastic aggregates concentrated after the evaporation step. The intermediate population at 226 nm is comparable in size to the dominant population of the milled PET reference suspension (291 nm, Figure 2A), supporting the use of the milled material as a reference for nanoplastic detection. The 226 nm population is also of similar magnitude to the Raman mapping grid step of 0.3 μm, again confirming the relevance of this spatial resolution for nanoplastic detection. The 32 nm population likely corresponds to salt aggregates, but from DLS, we cannot distinguish between these and nanoplastics at this size range, and their characterization would require techniques beyond the spatial resolution of conventional Raman spectroscopy.

The SERS measurements using the AgNP colloidal substrates were performed by drop-coating deposition Raman (DCDR). Unlike liquid colloidal SERS, where individual AgNPs interact with analyte particles in the suspension, the DCDR approach produces a different enhancement mechanism. During coffee-ring formation, the outward capillary flow concentrates AgNPs at the droplet periphery, resulting in dense nanoparticle packing approaching that of a solid silver film at the ring edge.^50^ PET nanoplastic particles concentrated at the ring edge under this same coffee-ring mechanism^51^ are therefore in closer proximity to multiple hotspot-forming interparticle gaps rather than to isolated individual nanoparticles as in liquid-phase SERS.

Nineteen mapping areas were systematically distributed at different positions of the coffee-ring region, based on optical microscopy images, from four independently dried droplets, to ensure the spatial representativeness of the extrapolation (Figure 3 and S2).

**Figure 3:**
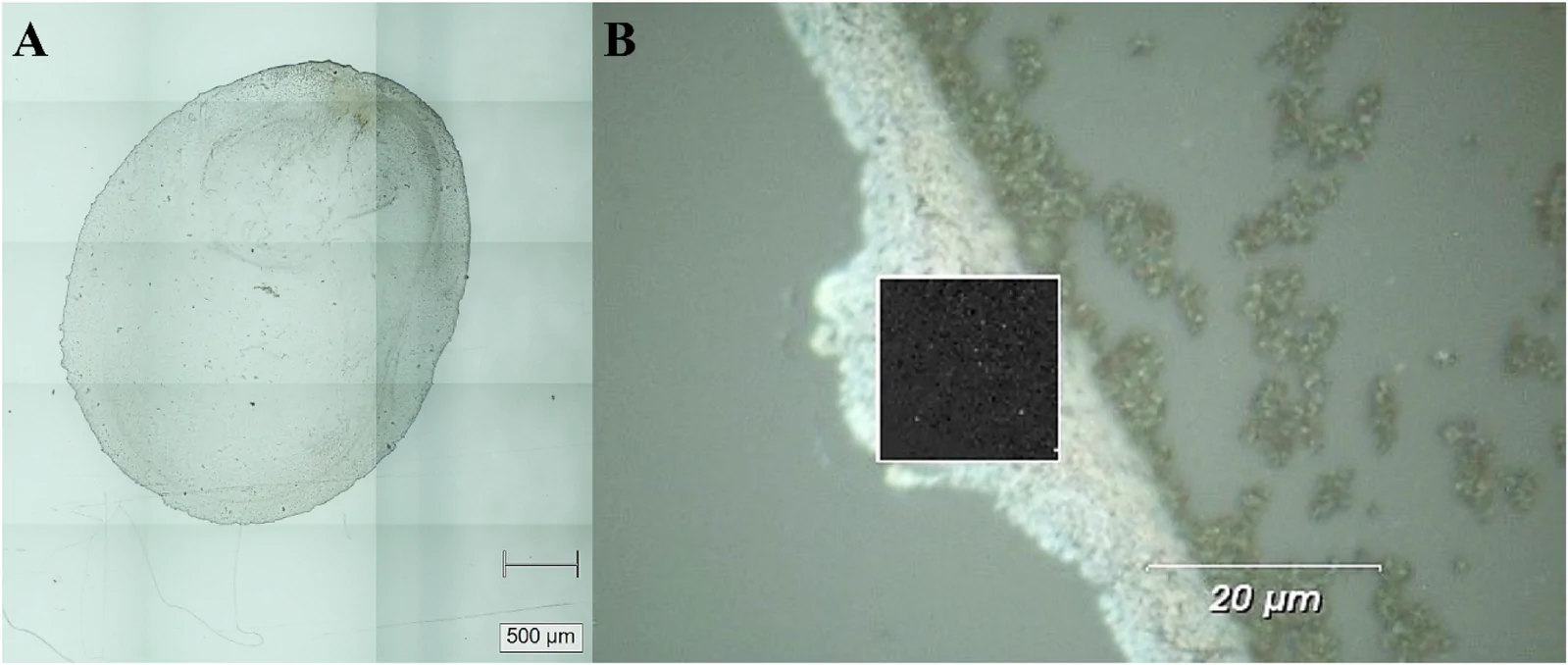
A) Optical microscopy image of one of the four dried 2 μL droplets containing concentrated bottled water residue mixed with colloidal silver nanoparticles. The image was created by fixing together nine individual photos acquired under a 5× objective. B) High-magnification image (100× objective) of the mapped region showing a grid of 0.3 × 0.3 μm^2^; white points indicate high spectral intensity in the 1690–1740 cm^–1^ region and serve as a preliminary visualization only of PET nanoplastics positions, final PET identification was performed by Pearson correlation analysis.

In Figure 4, six representative out of 19 mapping areas are shown as color-coded Pearson correlation coefficient maps overlapped onto the corresponding optical images. Regions with a Pearson coefficient above 0.7 indicate the highest possibility of PET nanoplastic presence. Two areas are further magnified (Figure 4, bottom), revealing 1 (left) and 2 (right) PET-positive particles within 544 μm^2^. The remaining mapped regions contained between 0 and 2 PET particles per area. No other polymer from the reference database (PE, PA, PVC, PP, and PS) produced positive identifications across any of the 19 mapped areas, consistent with the PET bottle being the primary source of nanoplastic in the sample.^12,52^ Identification was achieved by comparing spectra extracted from the maps with the reference PET spectrum from the Plastic Raman Spectra Database^41^ as shown in Figure 4. The detection threshold for PET nanoplastic identification was set at a Pearson coefficient of 0.7, confirmed by combining three approaches: spectral mixing with other polymers/pigments (Table S2), correlation with blank AgNP spectra, and artificial modification of the PET spectrum to mimic degradation (Figure S1). All three approaches converged on this value of 0.7, confirming it as a robust threshold.

**Figure 4:**
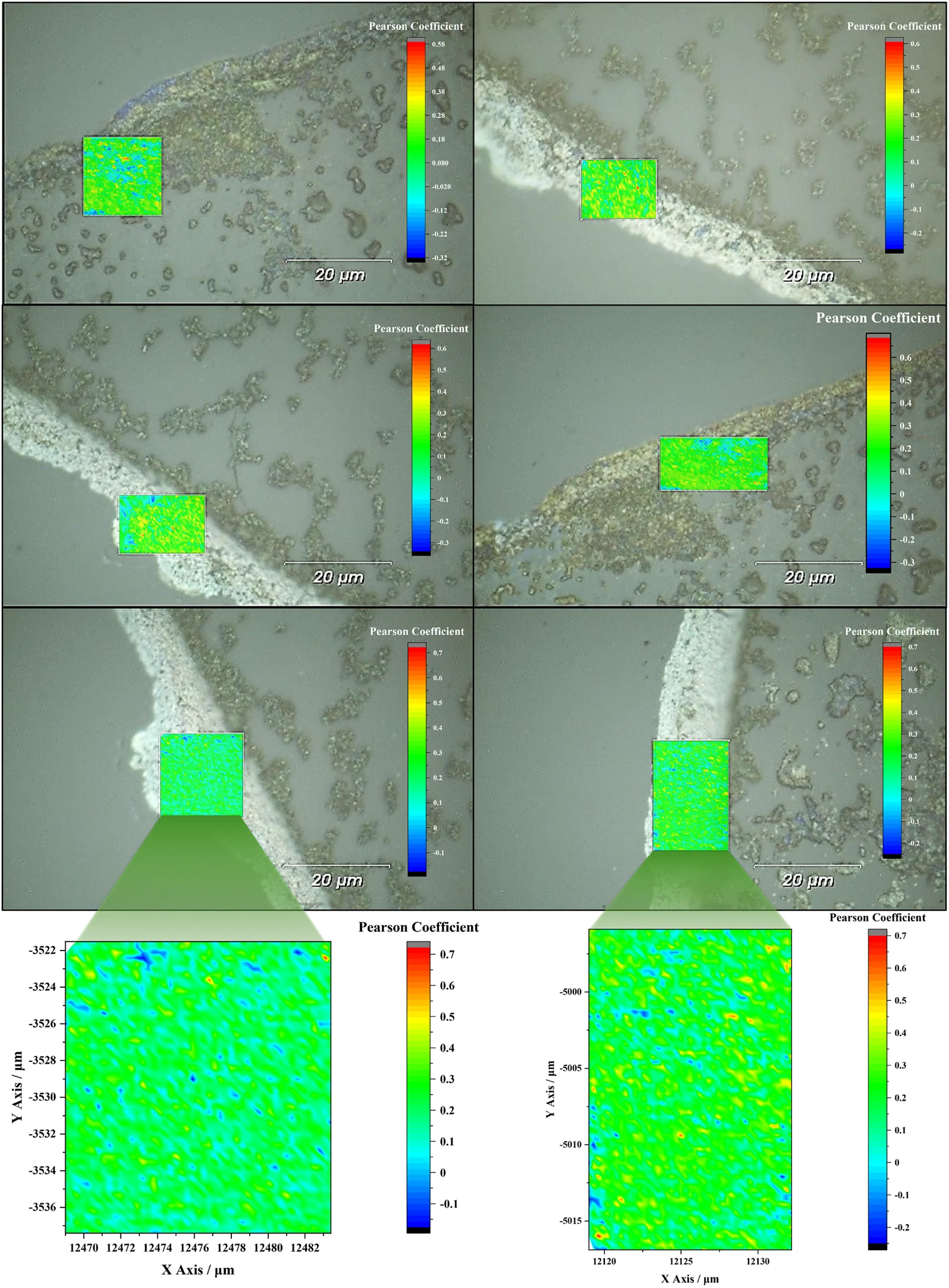
Spectral mapping results from silver colloid SERS measurements, generated by calculating the Pearson correlation coefficient between the reference PET spectrum and the measured Raman spectrum. Six representative mapping areas out of 19 are shown, overlaid on the corresponding optical microscopy images (100× objective, 532 nm laser excitation, 0.3 × 0.3 μm^2^ grid step, scale bars: 20 μm). Red regions indicate spatial locations with a high probability of PET nanoplastic presence (Pearson coefficient higher than 0.7). Bottom images show two magnified Pearson correlation maps revealing individual PET-positive mapping points at higher spatial detail.

Raman measurements performed using colloidal silver nanoparticles as the SERS substrate revealed complex spectral features, primarily due to signals originating from residual citrate from the silver nanoparticle synthesis (Figure 2B) as seen from milled PET SERS measurements. These signals are represented by intense and broad peaks that overlap with some characteristic plastic signals, possibly leading to false positives in polymer identification. A false-positive specificity evaluation was performed by applying an R > 0.7 threshold to approximately 15000 Raman spectra acquired from coffee-ring deposits of blank AgNP colloid containing no added nanoplastics, where zero spectra exceeded the threshold, confirming that citrate AgNP signals do not generate false-positive PET identifications (Figure S4).

To confirm that the algorithm has identified PET nanoplastics, the three diagnostic bands at 1287, 1614, and 1727 cm^–1^ must be present in the spectrum with a similar relative intensity as the reference PET spectrum (Figure 5). Considering the algorithm described above, the number of identified PET nanoplastics is 6 PET nanoplastics on an area of 2860 μm^2^ or 1 PET NP/477 μm^2^ (Figure S3). Each PET nanoparticle has an average area of 0.1 μm^2^, corresponding to one grid square in the Raman mapping, consistent with the particle size populations determined by DLS (Figure 2C).

**Figure 5:**
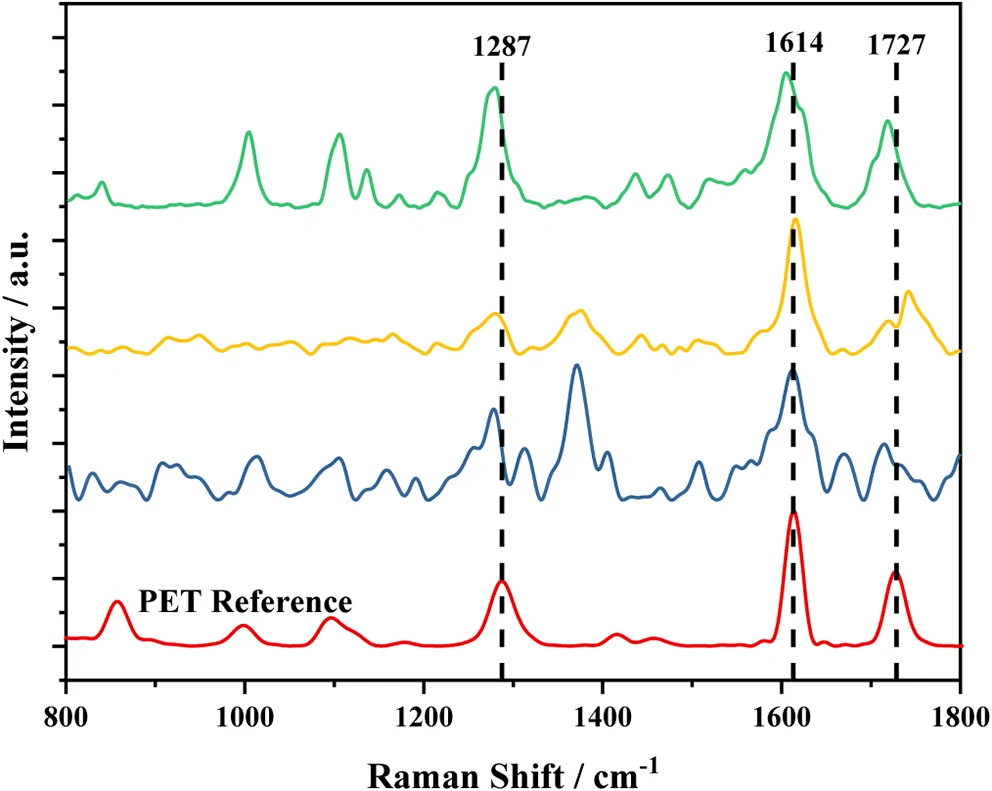
Representative (blue, yellow, and green lines) Raman spectra of PET nanoplastics identified in the bottled water sample using an AgNP—based SERS substrate (532 nm laser excitation) compared to the reference Raman spectrum of a PET fragment, as indicated. The key PET spectral bands at 1287 cm^–1^ (C–H bending), 1614 cm ^–1^ (aromatic C=C stretching), and 1727 cm ^–1^ (C=O stretching) are marked. Each identified spectrum exceeded the Pearson correlation threshold of R > 0.7 relative to the PET reference.

The quantification was based on calculating the area of the coffee ring in each dried droplet, following a similar spatial extrapolation used in microplastic and even nanoplastic quantification studies, where particle counts from a known subarea are extrapolated to the total sample area.^22,53–55^ Because concentration is derived from the spatial density of PET-positive points rather than from signal intensity, this approach does not require a signal-intensity calibration curve. The coffee-ring width was determined from optical microscopy images (Figure 3B and 4) as approximately 5 μm. Assuming the droplet is circular, the coffee-ring area was calculated as the difference between the total droplet area and the area of a smaller inner circle (excluding the ring). For the four measured dried droplets (Figure S2), the total coffee-ring area is 131475 μm^2^. Within this area, 276 PET nanoparticles were identified, corresponding to one particle per 477 μm^2^. Each dried droplet represents 2 μL of sample, so these 276 PET particles correspond to 8 μL of the AgNP-concentrated water mixture. Given the mixture ratio of 1/50 (evaporated water per AgNP colloid), 8 μL of mixture is equal to 0.16 μL of evaporated water. Scaling this value to the total evaporated volume of 10 mL, the estimated total number of PET nanoplastics in the original 0.5 L water bottle is approximately 17.25 million. Blank AgNP controls confirmed no background PET contamination (Figure S4). Based on the variability observed across the 19 mapped coffee-ring areas, the standard deviation (SD) of PET nanoparticle quantification was estimated to be approximately 1.676 million NPs or 9.5%. The SD was calculated as the square root of the variance, where the variance is the average of the squared differences between the PET counts in each mapped area and the mean PET count across all 19 areas. This variability reflects the spatial heterogeneity inherent to coffee-ring deposition and represents the dominant source of uncertainty in the reported particle count.

In addition to the detection of PET nanoplastics, the SERS analysis revealed spectral features similar to phthalocyanine (PhC) pigments (Figure 6). The color of these pigments shifts from blue to green depending on the presence or absence of atoms such as chlorine or bromine.^56,57^ Since these pigments share a common chemical structure with only minor structural variations, their Raman spectra exhibit small differences. Identifying such pigment particles gives valuable information about other types of contamination, especially those originating from packaging materials. The detection was based on characteristic Raman bands associated with a phthalocyanine specific band at 1531 cm^–1^, attributed to the C–C skeletal stretching mode of the phthalocyanine macrocycle.^58^ Additionally, data indicated an intense band or shoulder around 1333 cm^–1^, supporting the presence of phthalocyanine pigments. To confirm the source of these pigment particles, comparative Raman measurements were performed directly on the bottle cap and the bottle body (Figure 6).

**Figure 6:**
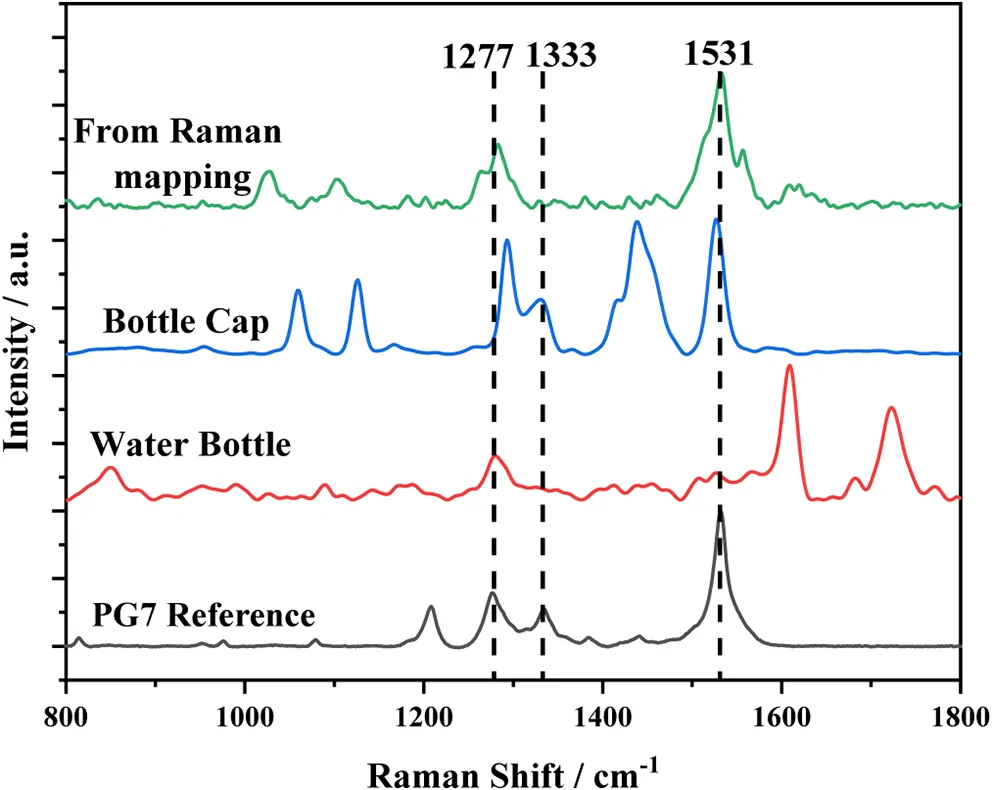
SERS identification of phthalocyanine green pigment (Pigment Green 7, PG7) in evaporated bottled water using colloidal silver nanoparticles as the SERS substrate. Raman spectra comparison: reference spectrum of PG7 (black line), classical Raman spectrum of the bottle body (red line), classical Raman spectrum of the bottle cap (blue line), and SERS spectrum extracted from Raman mapping of the water sample (green line). The characteristic PG7 bands at 1531 cm^–1^ (C–C skeletal stretching of the phthalocyanine macrocycle) and the shoulder at 1333 cm^–1^ are marked. Additional bands visible in the bottle cap spectrum originate from the polyethylene in which the phthalocyanine pigment is incorporated. Excitation wavelength: 532 nm.

The spectral comparison revealed that the PhC signature was present in the Raman spectrum of the bottle cap and was not present in the bottle body, which has an intense PET signal (Figure 6).

This finding suggests that phthalocyanine particles identified in the water sample originated from the bottle cap, likely being released during the opening and closing of the bottle. The additional Raman bands visible in the bottle cap spectrum beyond the phthalocyanine signature are attributed to the polyethylene matrix of the cap material in which the phthalocyanine pigment is incorporated as a colorant. However, a contribution from degradation of the bottle body cannot be excluded, considering that the bottle is light blue. It is important to note that minor spectral differences between the measured SERS signals and the reference phthalocyanine spectrum may arise from environmental degradation of the pigment or SERS enhancement; however, the contribution of the latter has been observed not to produce significant changes from the SERS studies on phthalocyanine.^59–61^

### SERS Measurements Using Commercial Solid Substrates

In addition to colloidal AgNPs, three commercial solid SERS substrates were evaluated for the analysis of the concentrated bottled water sample: Klarite, SERSitive, and μ-RIM. While the first two substrates include a noble metal (a raster of pyramids covered with gold in Klarite, and electrodeposited Ag nanoparticles on ITO glass in SERSitive, respectively), the hydrophobic μ-RIM provided only the stainless-steel surface. A small volume of 1 μL of concentrated bottled water was drop-coated on each substrate, and after drying, the analyses were performed using the laser excitation at 532 nm. The representative Raman spectra obtained are shown in Figure 7 with the corresponding reference spectra obtained from the RRUFF database.^62^ The Raman spectra revealed specific signals to calcite (calcium carbonate—CaCO_3_), gypsum (calcium sulfate dihydrate—CaSO_4_·2H_2_O), and sodium nitrate (NaNO_3_). A notable difference between the measured Raman spectrum of gypsum and the reference is the presence of a band at 1048 cm^–1^, which may originate from the presence of another carbonate mineral such as nahcolite (sodium bicarbonate—NaHCO_3_) whose Raman spectrum is characterized by a strong Raman band at 1045 cm^–1^. However, the presence of carbonate and sulfate minerals in bottled water is not surprising, as they commonly occur in dissolved form due to the natural ionic content of water from exploited spring sources. In our case, we were able to detect them because the sample preparation involved evaporating the water, which led to the formation of solid deposits containing these minerals and artificially increasing their concentration in the resulting evaporated solution.

**Figure 7:**
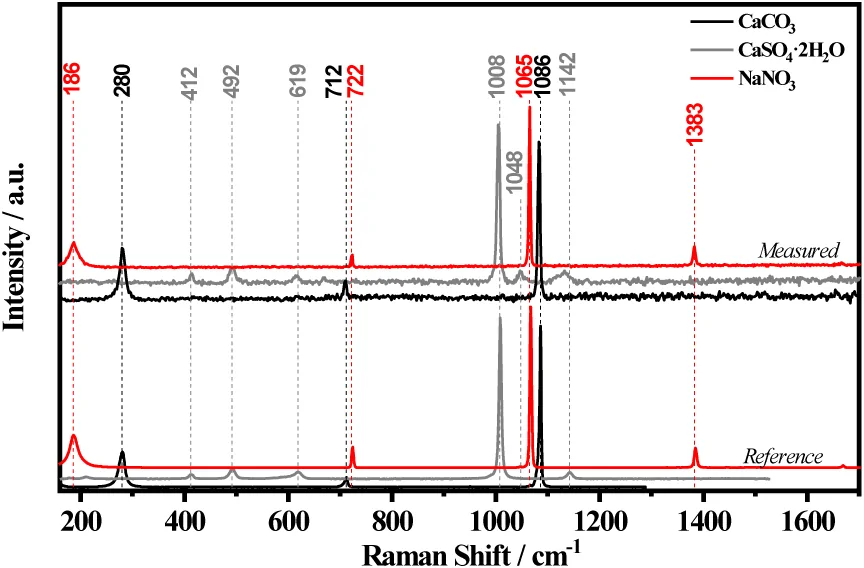
Raman spectra of inorganic salts (calcite, gypsum, and sodium nitrate) identified in the evaporated bottled water sample using all the three solid SERS substrates; reference spectra are sourced from the RRUFF database. Upper panel: measured spectra showing signals consistent with calcite (CaCO_3_, black), gypsum (CaSO_4_·2H_2_O, gray), and sodium nitrate (NaNO_3_, red). Lower panel: corresponding reference spectra. Excitation wavelength: 532 nm.

To detect nanoplastics in the concentrated bottled water with the commercial SERS substrates, we conducted mapping measurements on each substrate. Among the commercial substrates tested, only Klarite generated SERS signals representative for PET nanoplastics. The mapping area is shown in Figure 8A, and the obtained SERS spectra are shown in Figure 8B. The presented signals correspond to polyethylene terephthalate (PET) and were identified using the same automated spectral analysis as in the case of the colloidal substrate. From the three PET diagnostic bands, only 1287 and 1614 cm^–1^ are better resolved in the Klarite spectra (Figure 8B), while the 1728 cm^–1^ band, being the weakest of the three by intensity, is drowned out by the background signal from the deposited inorganic salts, which add noise across the Raman spectrum. The additional band around 1050 cm^–1^ is indicating again the presence of a carbonate mineral in the water sample, consistent with nahcolite (NaHCO_3_) previously identified in the evaporated water sample (Figure 7). However, these findings confirm the presence of nanoplastics in bottled water, and the likely source of these nanoplastic particles is the bottle itself, made of PET.

**Figure 8:**
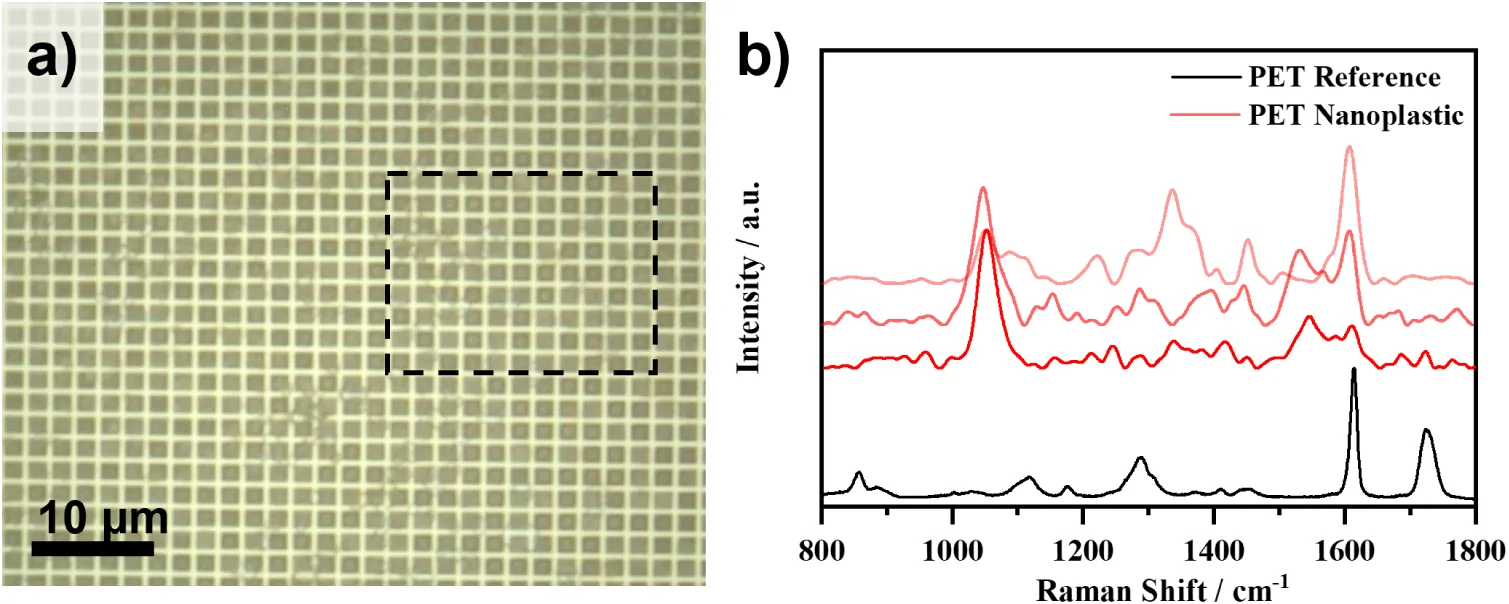
A) The microscopic image of the mapping area on the Klarite substrate with a 100× objective, showing the characteristic gold inverted pyramid nanostructure array; the dashed rectangle indicates the Raman mapping area (10 × 10 μm^2^, 0.7 μm grid step, scale bar: 10 μm), and B) three representative SERS spectra of PET nanoplastics (red line) and the Raman spectrum of PET plastic (black line) as reference with the diagnostic PET bands at 1287 and 1614 cm^–1^ are resolved. Excitation wavelength: 532 nm.

### SEM-EDX Characterization of Nanoplastics in Bottled Water

Scanning electron microscopy (SEM) coupled with energy dispersive X-ray spectroscopy (EDX) was used to investigate the morphology and elemental composition of particles in the concentrated bottled water sample. SEM imaging revealed nanoscale particles with irregular shapes and sizes (Figure 9). EDX analyses confirmed that these particles were primarily composed of carbon (C) and oxygen (O), with C/O ratios varying across different particles. Particles showing C/O ratios near 65% C and 35% O are indicative of PET composition, matching its stoichiometry (Figure 9B). However, some particles have higher carbon content, which may result from thermal degradation of some organic material during sample evaporation or from other types of nanoplastics such as PE and PP, though oxidation may have changed their Raman signatures beyond detection (Figure 9D). Although EDX analysis confirms the presence of carbon and oxygen at ratios consistent with PET stoichiometry, it lacks molecular specificity as other organic particles can have a similar C/O ratio. Therefore, these results serve as complementary evidence and need validation from the SERS analysis.

**Figure 9:**
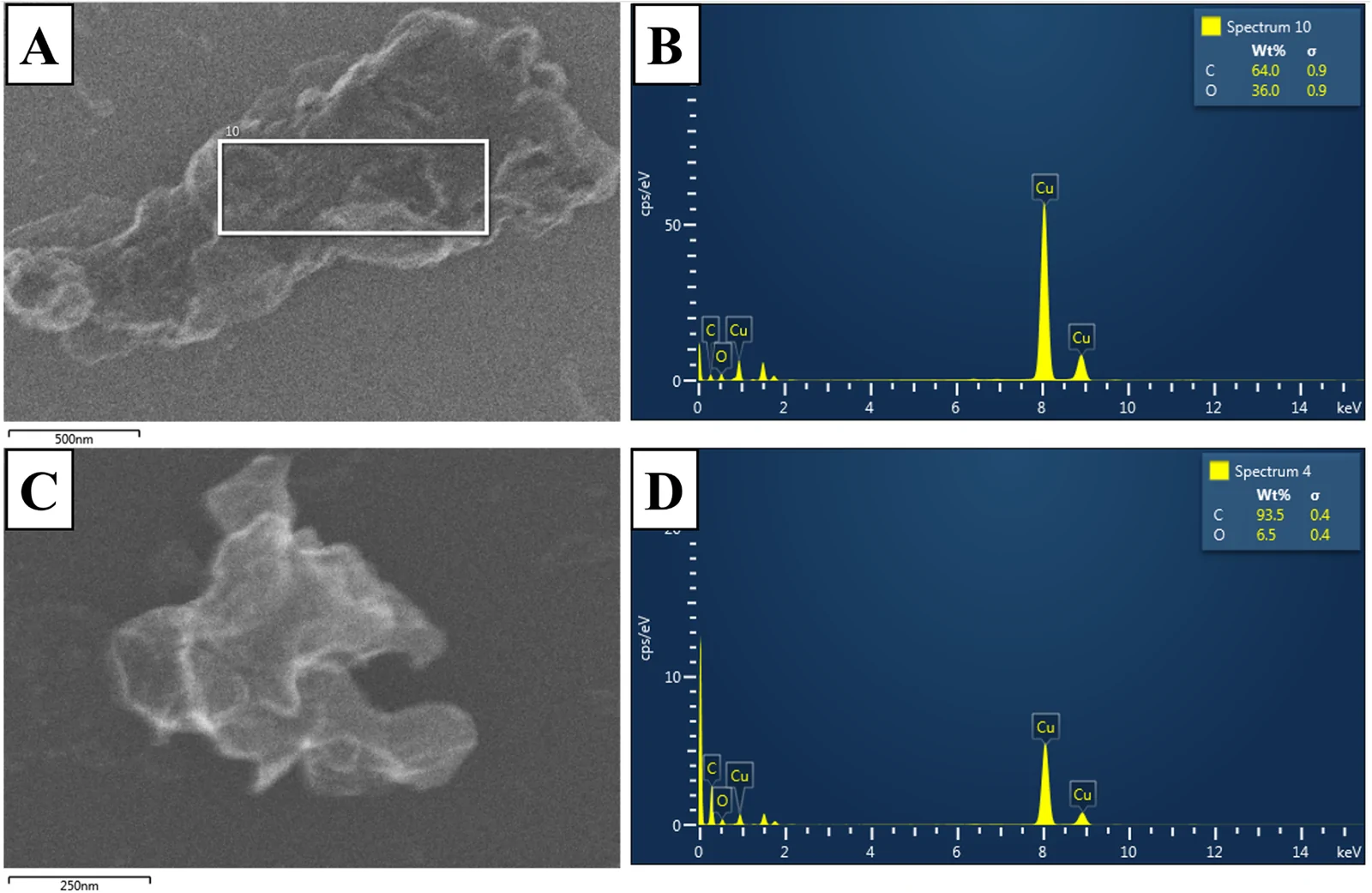
Representative Scanning Electron Microscopy (SEM) images (A and C) and Energy-Dispersive X-ray (EDX) elemental analyses (B and D) of the concentrated bottled water sample after evaporation. The SEM images reveal nanometer-sized particles, while the EDX spectra show variations in the carbon-to-oxygen (C/O) ratio. In image C, the C/O ratio is consistent with PET nanoplastics. In contrast, image D shows particles with different elemental compositions, which may indicate degraded organic material formed during evaporation, carbon-rich particles naturally present in the water or possibly oxidized polyethylene or polypropylene nanoplastics.

## Discussion

By using natural water from commercial bottles produced for market, we demonstrated the applicability of the SERS technique for detecting and characterizing nanoplastics in aqueous environments without adding them intentionally, as previously reported in many SERS studies aimed to detect and quantify nanoplastics.^22,53–55^ The use of colloidal AgNPs in the SERS method allows the detection of PET nanoplastics, along with phthalocyanine pigments likely originating from tiny particles from a cyan bottle cap. The challenges in correct analyses are due to background contributions either from dissolved salts in water (as in the case of solid SERS substrates) or, in the case of colloidal measurements, from silver nanoparticle aggregates. These limitations highlight the need for more advanced and automated computational techniques to enable faster and more validated algorithms for large Raman data sets. In addition, the detection of PET signals in only 5 out of 19 Raman mappings suggests that improvements in sample preparation and measurement strategy are necessary. However, further concentration through extended evaporation can promote uncontrolled AgNP aggregation from a high concentration of salts.^63^ Regarding measurement strategy, increasing either the number of mapped areas or the total mapped area would improve the statistical confidence of the particle count, with the primary constraint being the acquisition time required per mapping.

The complementary use of commercial solid SERS substrates provided advantages such as reduced interference from colloidal aggregation. Among the tested substrates, Klarite showed the highest performance, successfully detecting PET nanoplastics, though in lower numbers compared to those of colloidal AgNPs. This difference can be explained by the coffee-ring effect: during evaporation, AgNPs move with PET nanoparticles toward the droplet edge, artificially increasing the concentration at the margins while decreasing the concentration at the center.^64^

To visualize this spatial distribution directly, a Pearson correlation map was acquired over the full dried droplet area using a 5× objective (NA = 0.12, spatial resolution = 2.2 μm at 532 nm laser excitation) and a 10 × 10 μm mapping grid (Figure 10). The spatial distribution of Pearson coefficient values shows a clear peripheral gradient, with higher correlation coefficients concentrated at the droplet edge and systematically lower values in the interior, providing direct spatial evidence of coffee-ring-driven particle accumulation. The maximum Pearson coefficient across the full droplet map (0.644) does not reach the 0.7 identification threshold because the grid step averages the PET signal with the AgNP-citrate background over each 100 μm^2^ measurement area, confirming that the high-resolution 0.3 μm coffee-ring mapping is essential for identification of individual nanoplastics.

**Figure 10:**
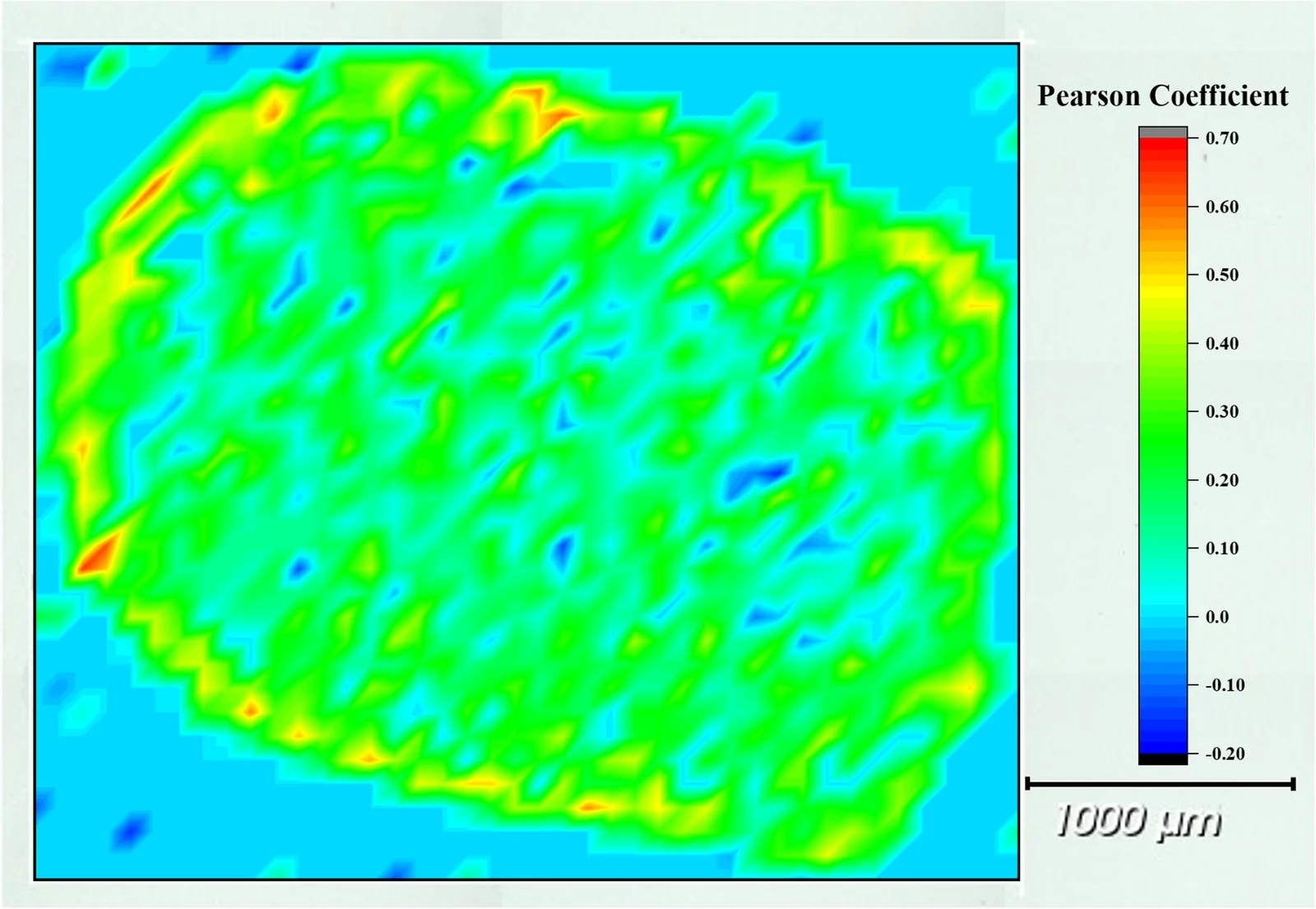
Pearson correlation map of the entire dried droplet area compared to the PET reference Raman spectrum, acquired using a 5× objective with a 10 × 10 μm grid step. Higher Pearson coefficient values (red) are preferentially localized at the droplet periphery, confirming coffee-ring-driven concentration of PET nanoplastics at the ring edge.

Additionally, the identification of phthalocyanine pigments indicates that bottled water contamination extends beyond polymer nanoparticles, also being present secondary additives that may be a cause for additional health risks. PET bottles are known to release such additives, including plasticizers, antioxidants, thermal stabilizers, and colorants, into their contents with phthalate esters reported at 0.19–0.98 μg/L in PET-bottled water under standard storage conditions and up to 150 chemicals identified as migrants from PET packaging.^65,66^ Although quantification and health risk assessment of such additives were beyond the scope of this study, the simultaneous detection of phthalocyanine pigment particles alongside PET nanoplastics shows that the proposed method is sensitive enough to reveal additive contamination as part of the nanoplastic screening protocol, representing a direction worth pursuing in future work.

Furthermore, the detection of inorganic minerals such as calcite, gypsum, and sodium nitrate—naturally occurring components of drinking water—shows that SERS can also be applied to salt-level monitoring with minimal sample preparation, as highlighted within an independent study, using the same AgNPs.^67^

From a quantification perspective, based on colloidal AgNP measurements, the estimated density of PET nanoplastics was 1 particle/477 μm^2^. Extrapolating to the 0.5 L water sample analyzed, this corresponds to approximately 17.25 million PET nanoparticles per bottle, a value comparable to concentrations reported in other studies based on the quantification of PET nanoplastics in bottled water.^38,68^ This particle number concentration, derived directly from counting Pearson-positive detection events without assumptions about particle morphology, is the primary quantification metric used in this study. To facilitate comparison with literature data reported in mass units and considering the mean hydrodynamic diameter of the nanoplastic DLS population (226 nm) and the bulk PET density of 1.38 g/cm^3^, this particle concentration corresponds to an estimated mass concentration of approximately 288 ± 27 ng/L.

However, it is important to highlight that these values likely represent approximate estimates. The nanoplastics identified in this study were predominantly nondegraded PET fragments. Chemically, mechanically, or photochemically changed PET polymer may exhibit a different Raman signature, which means a lower Pearson threshold, increasing the probability that more highly degraded particles remained undetected or were not confidently classified due to these spectral modifications.^38,69,70^

From a methodological perspective, the quantification approach used here is based on spatial particle counting, following the same principle established in nanoplastic and microplastic quantification studies.^22,53–55^ Because the concentration is derived from the spatial density of PET-positive points rather than from signal intensity, this approach does not require a calibration curve. The validity of the detection algorithm is supported by the milled PET reference experiment (Figure 2), which confirmed the correct identification of real PET nanoparticles under identical experimental conditions. Liquid SERS measurements, while applicable because of speed of the results, offer limited practical advantage for hydrophobic polymers such as PET, whose weak affinity for citrate-capped AgNP surfaces reduces the probability of molecule-nanoparticle interaction.^71,72^ The drop-coating approach solves this by concentrating both nanoplastics and AgNPs at the coffee-ring during drying, forcing their interaction and additionally permitting simultaneous identification of multiple molecules, such as PET nanoplastics, phthalocyanine pigments, and inorganic residues from a single dried droplet, which liquid measurements cannot achieve with comparable efficiency.^64^

Altogether, these findings support the potential of SERS as a versatile and sensitive technique for the detection, quantification, and monitoring of nanoplastics in drinking water and other aqueous matrices.

## Conclusions

This study demonstrates that SERS, supported by plastic identification algorithms, is a promising tool for the detection and characterization of PET nanoplastics in bottled water. Comparing colloidal AgNPs with commercial solid substrates, the results indicate that colloidal SERS provides higher sensitivity, but still additional spectral bands from aggregation complicate signal interpretation. These challenges, however, can be overcome through computational analysis. The confirmed presence of PET and phthalocyanine pigment nanoparticles highlights multiple contamination sources originating from packaging materials, validating greater attention to the potential risks posed by these particles. Furthermore, while the detection of salts is less remarkable from a spectroscopic perspective, it still accentuates the versatility and broader applicability of SERS in water quality analysis.

## Supplementary Material



## Notes

PET Raman spectrum modified to simulate degradation effects; Pearson correlation coefficients between PET and reference pigment/plastic spectra; Pearson correlation coefficients for computationally mixed PET-pigment spectra; optical microscopy images of dried droplets; SERS spectra of all identified PET nanoplastics across mapping areas; and Pearson correlation maps of blank AgNP controls (PDF)
