# Supplementary material for: SERS of Bottled Water: Automated PET Nanoplastic Detection and Quantification
Source: Anal Chem. 2026 Sep 26;98(43):29956–69. doi: 10.1021/acs.analchem.6c04234 (PMC13738000; doi:10.1021/acs.analchem.6c04234)
Supplement: Supplementary file 1 [file ac6c04234_si_001.pdf]

Supplementary Information

SERS of bottled water: automated PET nanoplastics detection and quantification

Ion Nesterovschi [1, 2, 3*], Ioana Cârdan [1, 2, 3], Karlo Maškarić [1, 2, 3], Anamaria Couți [1,2], Lucian Barbu-Tudoran [3], Simona Cinta-Pinzaru [1,2 *]

1. Biomolecular Physics Department, Babeş-Bolyai University, Kogălniceanu 1, RO-400084, Cluj-Napoca, Romania
2. Institute for Research, Development and Innovation in Applied Natural Sciences, Babes-Bolyai University, Fântânele 30, RO-400327 Cluj-Napoca, Romania
3. National Institute for Research and Development of Isotopic and Molecular Technologies, Cluj-Napoca, 400293, Romania

Table of Contents

Figure S1. Changed PET Raman spectrum to mimic spectral changes under degradation ... S2

Table S1. Pearson correlation coefficients between PET reference spectrum and pigment/plastic reference spectra …………………………………………………………...... S2

Table S2. Pearson correlation coefficients between PET spectrum and computationally mixed PET-pigment spectra at increasing intensity ratios ……………………………………......... S2

Figure S2. Optical microscopy images of dried droplets used for SERS measurements S3

Figure S3. SERS spectra of all PET nanoplastics identified across 19 Raman mapping areas S4

Figure S4. Pearson correlation maps of blank AgNP control samples S4

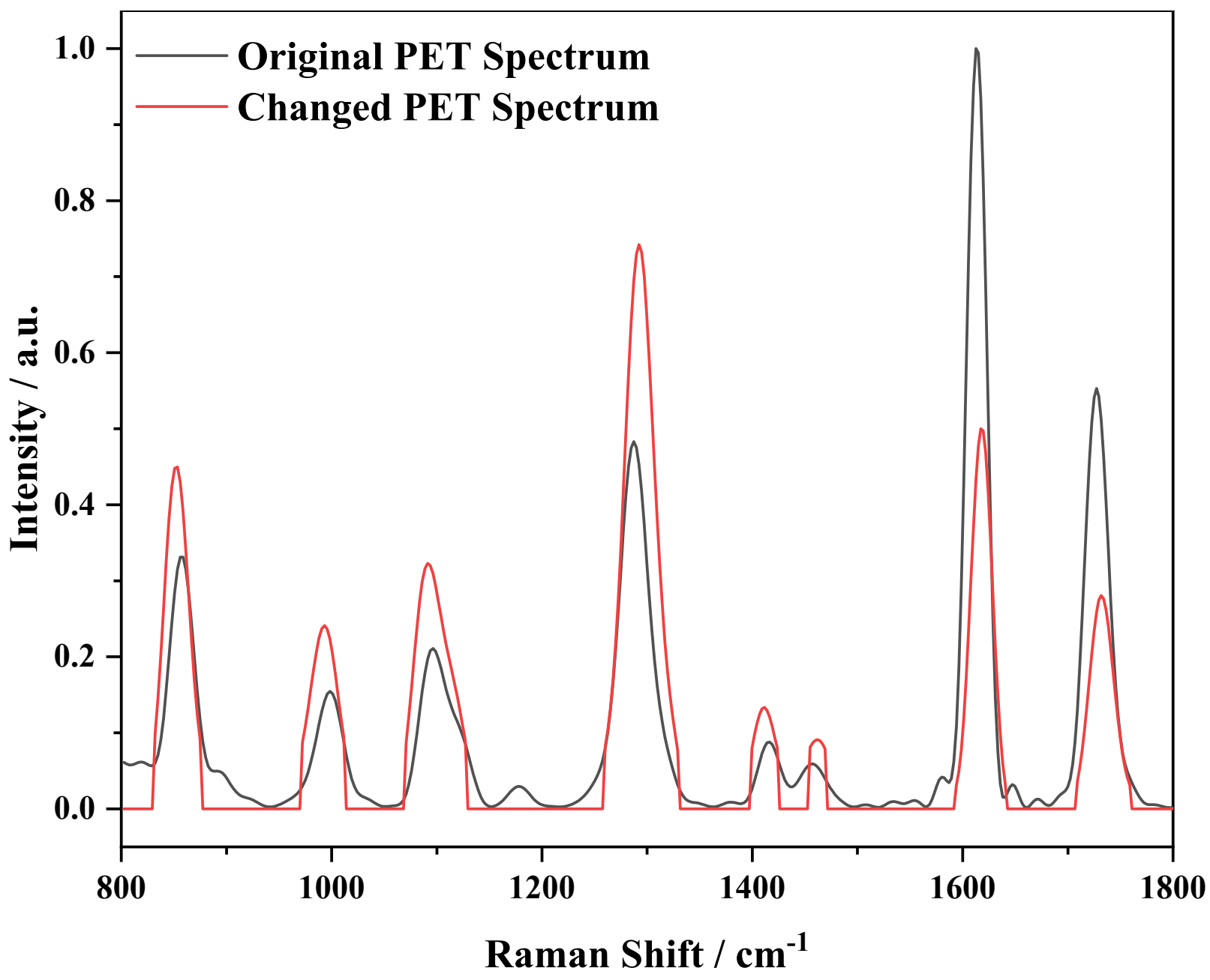

Figure S1. Changed PET Raman spectrum to mimic spectral changes under degradation, by changing the intensities of the bands by 50% and to shift their position by 5 cm^{-1}. Pearson coefficient between original PET spectrum and the changed one is 0.71.

Table S1. The comparison results between PET Raman spectrum to the pigments and plastics from the Raman databases [1,2] by Pearson correlation coefficient in descending order, which highlights the robustness of the threshold of 0.7.

Pigment name	Pearson coefficient	Pigment name	Pearson coefficient	Pigment name	Pearson coefficient
Pigment Green 1	0.522	Pigment Yellow 210	0.391	Pigment Yellow 94	0.313
Pigment Blue 62	0.487	Pigment Violet 17	0.382	Pigment Red 226	0.307
Pigment Yellow 214	0.406	Pigment Yellow 219	0.379	Pigment Yellow 6	0.305
Cellulose Acetate	0.406	Pigment Yellow 169	0.339	Pigment Orange 13	0.299
Pigment Violet 5	0.403	Pigment Blue 24	0.336	Pigment Yellow 3	0.275

Table S2. Pearson correlation coefficients between the PET reference Raman spectrum and computationally mixed spectra of PET combined with two pigments (PB15 and PR260) at increasing pigment-to-PET intensity ratios (0.2 to 2.0).

Pigment mixture	Pigment / PET intensity ratio	Pearson coefficient	Pigment mixture	Pigment / PET intensity ratio	Pearson coefficient
PET + PB15	0.2	0.892	PET + PR260	0.2	0.907
PET + PB15	0.4	0.880	PET + PR260	0.4	0.908

PET + PB15	0.6	0.861	PET + PR260	0.6	0.908
PET + PB15	0.8	0.818	PET + PR260	0.8	0.915
PET + PB15	1.0	0.797	PET + PR260	1.0	0.914
PET + PB15	1.2	0.757	PET + PR260	1.2	0.908
PET + PB15	1.4	0.715	PET + PR260	1.4	0.913
PET + PB15	1.6	0.672	PET + PR260	1.6	0.911
PET + PB15	1.8	0.630	PET + PR260	1.8	0.909
PET + PB15	2.0	0.589	PET + PR260	2.0	0.907

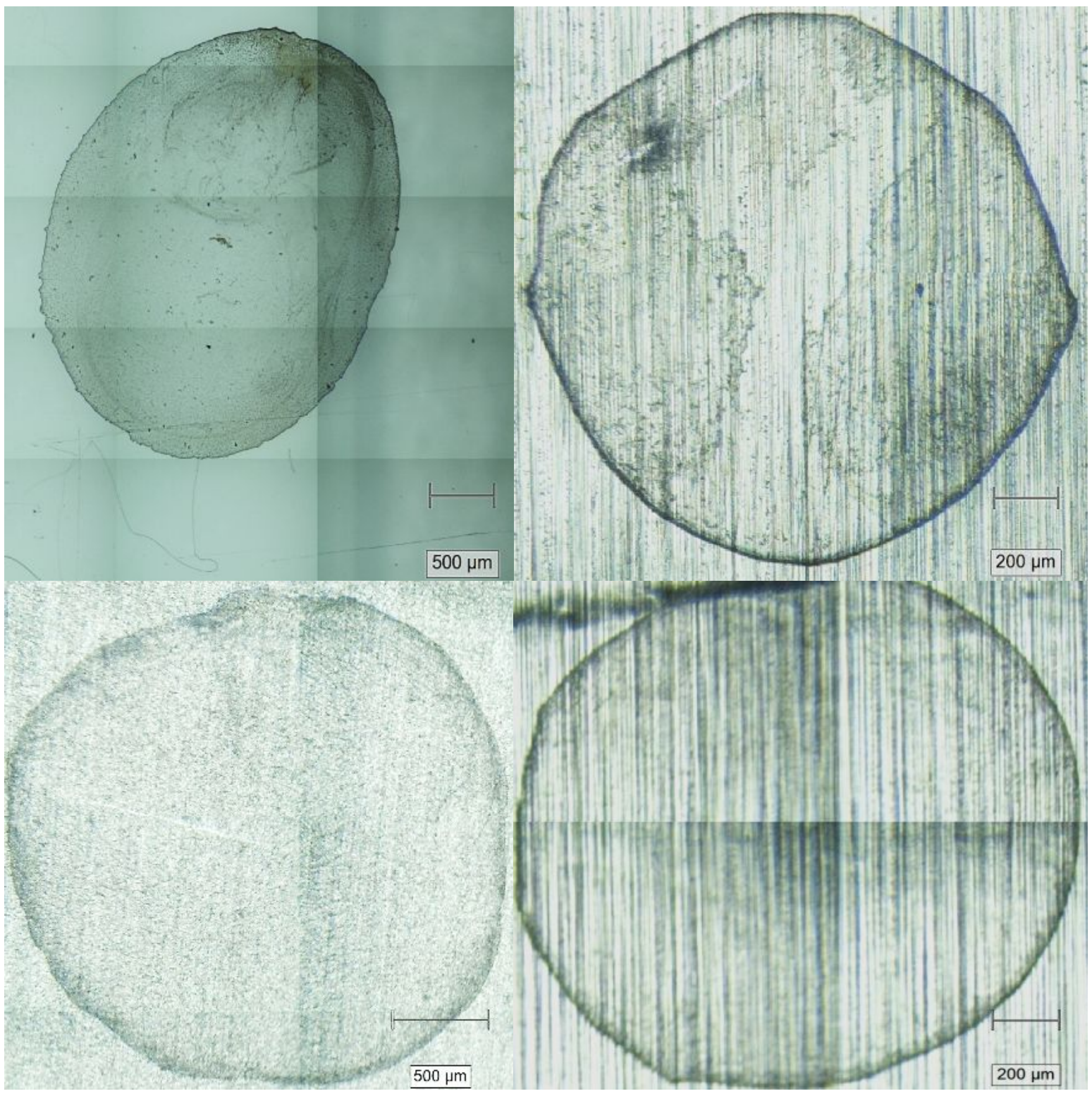

Figure S2. All dried drops from the mix of colloidal silver with the evaporated bottled water on the aluminium foil used for SERS measurements. Objective 5X.

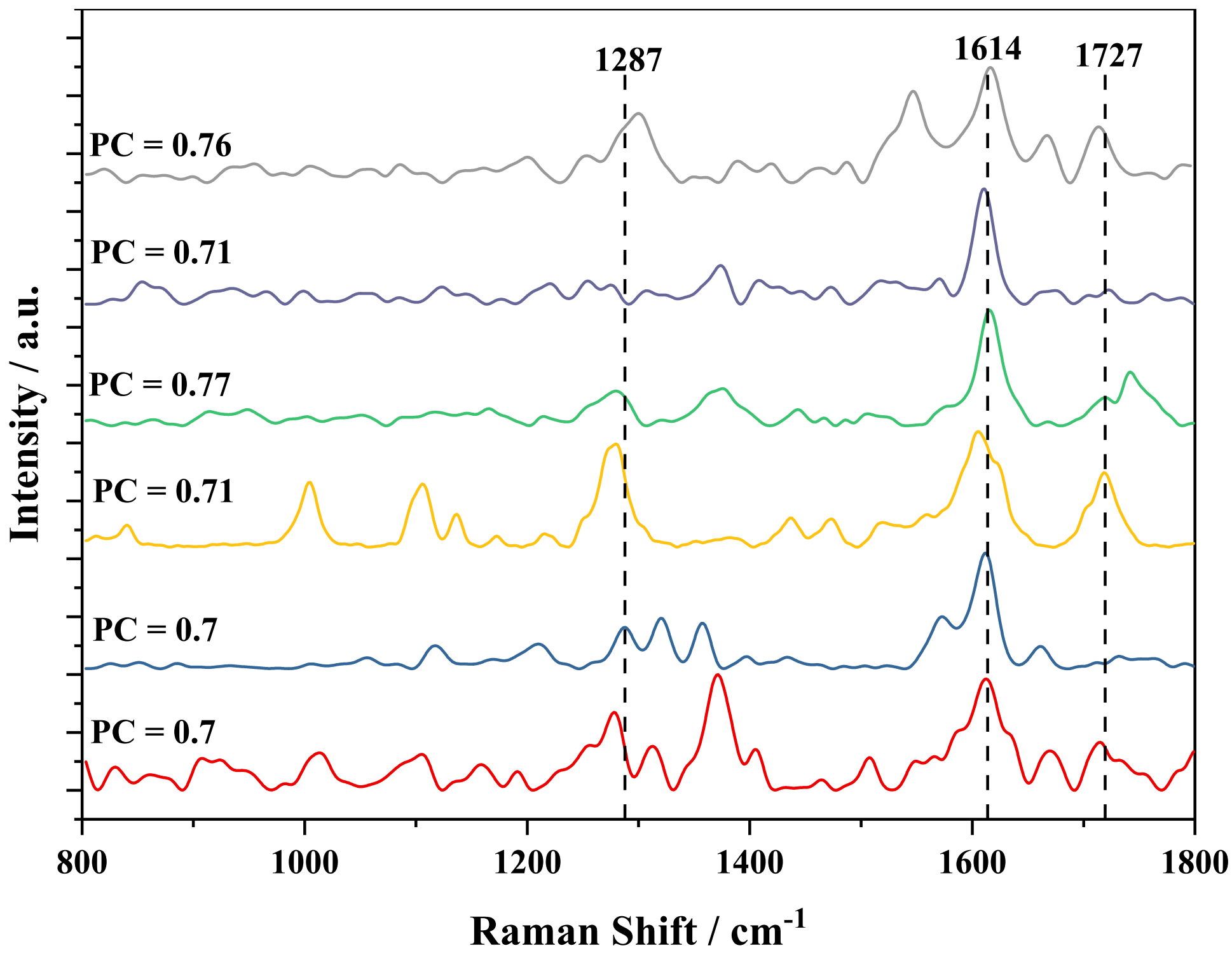

Figure S3. SERS spectra of all PET nanoplastics identified across 19 Raman mapping areas of the evaporated bottled water sample using colloidal AgNPs with corresponding Pearson correlation coefficient (PC) to PET reference Raman spectrum. The characteristic PET bands at 1287 cm^{-1}, 1614 cm^{-1} and 1727 cm^{-1} are indicated. Band shifts between spectra are a result of environmental degradation. Excitation wavelength: 532 nm.

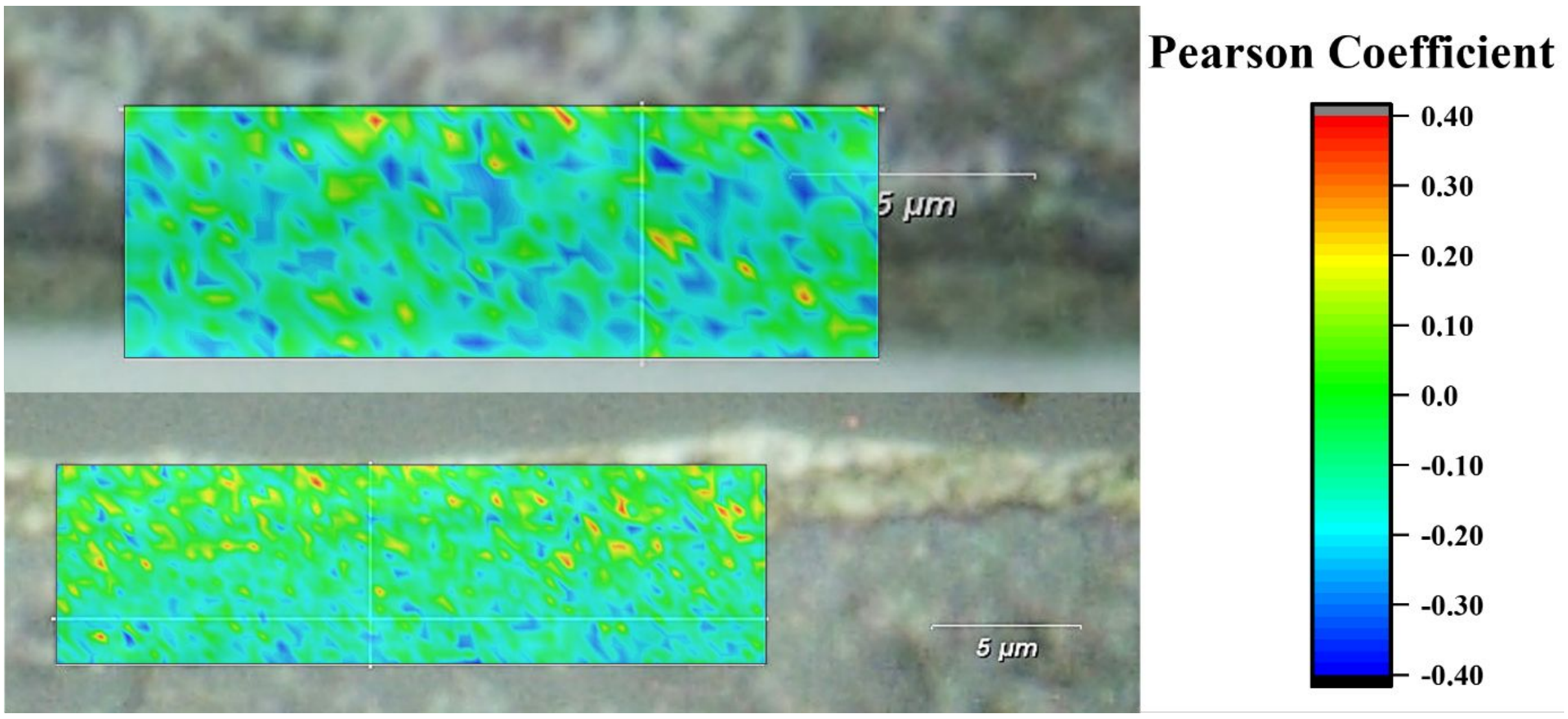

Figure S4. Pearson correlation maps from two representative Raman mapping acquisitions of blank AgNP-distilled water dried droplets containing no added nanoplastics. No pixel in either map exceeds the R = 0.7 identification threshold, confirming the absence of false-positive PET identifications arising from the citrate-capped AgNP substrate under the measurement conditions used. Scale bar: 5 µm. Excitation wavelength: 532 nm.

References:

(1) Marica, I.; Pînzaru, S. C. A Raman Spectral Database of Naturally Aged Plastics: A Proof-of-Concept Study for Waste Plastic Sorting. *Journal of Raman Spectroscopy* **2023**, *54* (3), 305–313. https://doi.org/10.1002/JRS.6484;WGROUP:STRING:PUBLICATION

(2) Fremout, W.; Saverwyns, S. Identification of Synthetic Organic Pigments: The Role of a Comprehensive Digital Raman Spectral Library. *Journal of Raman Spectroscopy* **2012**, *43* (11), 1536–1544. https://doi.org/10.1002/JRS.4054
